# Effect of Cytochrome P450 2C9 genetic polymorphism on agomelatine metabolism* in vitro*

**DOI:** 10.7717/peerj.20973

**Published:** 2026-03-19

**Authors:** Ping Fang, Jinhuan Ni, Taoye Shen, Qing Chen, Xinwu Ye, Jia Jin, Dapeng Dai, Guoxin Hu, Jianchang Qian, Jianping Cai, Lianguo Chen

**Affiliations:** 1 Department of Clinical Pharmacy, The First Affiliated Hospital, Zhejiang University School of Medicine, Hangzhou, Zhejiang, China; 2 Department of Pharmacology, Wenzhou Medical University, Wenzhou, China; 3 Department of Psychiatry, Wenzhou Seventh People’s Hospital, Wenzhou, China; 4 The Key Laboratory of Geriatrics, Beijing Hospital & Beijing Institute of Geriatrics, Beijing, China; 5 Department of Pharmacy, The First Affiliated Hospital of Wenzhou Medical University, Wenzhou, China

**Keywords:** CYP2C9, Genetic polymorphism, Recombinant microsomes, Agomelatine, UPLC-MS/MS

## Abstract

**Background:**

Genetic polymorphisms of CYP2C9 significantly influence the efficacy and safety of some drugs, which potentially lead to adverse effects and therapeutic failure. The aim of this study was to investigate the genetic polymorphism of 37 CYP2C9 alleles and evaluate their catalytic activities in agomelatine metabolism *in vitro*.

**Methods:**

Insect microsomes expressing the 37 recombinant CYP2C9 variants were incubated in a 37 °C water bath with agomelatine. The main active metabolites of agomelatine were detected using a UPLC-MS/MS system. Then, the enzyme kinetic parameters of the 36 variants were calculated and compared with those of the wild-type CYP2C9*1.

**Results:**

Relative to CYP2C9*1, four variants exhibited no significant difference in enzyme activity. Eleven variants exhibited significantly higher intrinsic clearance values, while 13 variants exhibited significantly reduced intrinsic clearance values. The remaining eight variants demonstrated complex metabolic patterns; these variants do not produce the two metabolites equally, and they inhibited the appearance of one metabolite but promoted the production of another metabolite. These results suggest that special attention should be given to subjects carrying poor metabolizers of *CYP2C9* alleles when prescribing agomelatine in clinical practice. This study can provide valuable insights for personalized medicine and reduce adverse reactions to some extent.

## Introduction

Agomelatine (AGO), a specific agonist of melatonin receptors (MT1 and MT2) and a selective antagonist of the serotonin 2C (5-HT2C) receptor, is a relatively new antidepressant with a different mechanism of action than other antidepressants (Owen, 2009; Smeraldi & Delmonte, 2013). As such, it could be a valuable additional treatment option for patients who exhibit an inadequate response to other antidepressants or cannot tolerate the side effects of them. The most common adverse reactions associated with agomelatine include headache, nasopharyngitis, gastrointestinal discomfort and elevated liver transaminases (Owen, 2009; Dolder, Nelson & Snider, 2008; Gao et al., 2023; Wang et al., 2021). Given the relatively high incidence of hepatotoxic reactions, regular laboratory monitoring of liver function is recommended throughout the course of agomelatine treatment (Howland, 2011). However, patient-specific risk factors associated with agomelatine-induced liver injury remain unclear. The hepatotoxicity of agomelatine may be related to individual metabolic responses or genetic profiles (Jia et al., 2019).

**Figure 1:**
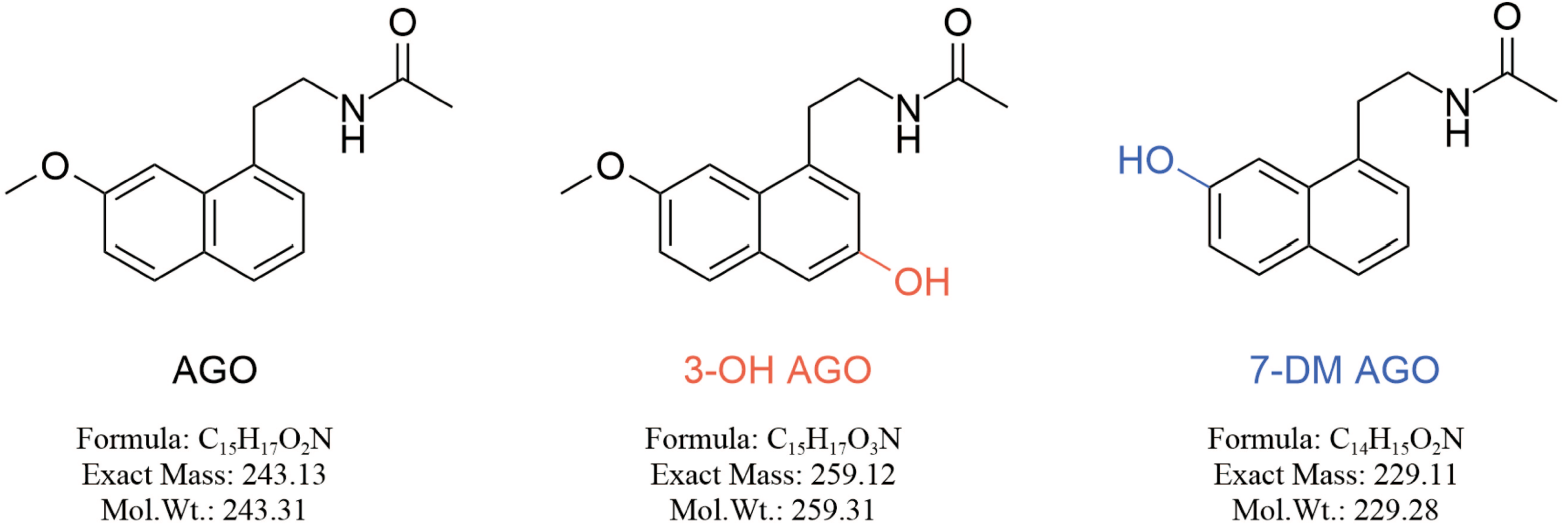
Chemical structural formula of AGO and its metabolites (3-OH AGO and 7-DM AGO).

Genotypic polymorphisms of cytochrome P450 (CYP450) superfamily often led to large inter-individual differences in the rate and route of drug metabolism. Agomelatine is extensively metabolized *via* CYP450 isoenzymes CYP1A2, and CYP2C9 into two metabolites (Fig. 1), 3-hydroxy agomelatine (3-OH AGO) and 7-desmethyl agomelatine (7-DM AGO), both of which exhibit lower pharmacological activity compared to agomelatine (Dolder, Nelson & Snider, 2008; Sansone & Sansone, 2011). CYP1A2 phenotype inferred from the genotyping of CYP1A2*1C, *1F and *1B alleles might be a potential predictor of agomelatine exposure (Owen, 2009). Agomelatine is contraindicated in patients with impaired liver function or in those taking concomitant drugs that strongly inhibit CYP1A2, due to the risk of increased systemic exposure (Howland, 2011). Notably, defective alleles of *CYP2C9* were more common in Asian populations, and individuals with decreased enzyme activity of CYP2C9 may also result in higher blood concentration of agomelatine and associated adverse reactions (Prasertsup et al., 2023; Silvado, Terra & Twardowschy, 2018; Thakkar et al., 2012). A previous report have documented that polymorphisms in *CYP2C9* significantly affect agomelatine pharmacokinetics in healthy volunteers, CYP2C9 intermediate/poor metabolizers exhibited higher area under the concentration–time curve (AUC) and maximum concentration(C_max_) (Saiz-Rodríguez et al., 2019). Twenty-two novel alleles of *CYP2C9* have been identified in our previous study, but the functional impact of these newly discovered variants on the metabolism and clinical toxicity of agomelatine remains to be elucidated (Hu et al., 2015; Dai et al., 2013).

In our previous research, the inhibitory effect of celecoxib on agomelatine metabolism in rats was demonstrated (He et al., 2018). The main role of CYP2C9 in the depletion and oxidative metabolism of celecoxib has been confirmed based on inactivated human liver microsomes (Murayama et al., 2018). Our *in vitro* results indicated that celecoxib inhibits CYP2C9-mediated agomelatine metabolism. Thus, we speculate that changes in CYP2C9 activity may affect the pharmacokinetics of agomelatine.

In this study, we systematically analyzed the catalytic activities of CYP2C9*1 and 36 variant alleles (*2, *3, *8, *11, *13, *14, *16, *19, *23, *27, *29, *31, *33, *34, *36, *38, *39, *40, *41, *42, *43, *44, *46, *47, *48, *49, *50, *51, *52, *53, *54, *55, *56, *58, *59, *60) found in the Chinese population toward agomelatine *in vitro*. Our findings aim to provide valuable information for further studies on *CYP2C9* alleles for agomelatine metabolism and personalized dosing strategies in clinical practice.

## Materials & Methods

### Chemicals and materials

Agomelatine, 7-desmethyl-agomelatine and 3-hydroxy-agomelatine were purchased from Tokyo Chemical Industry Co., Ltd (Tokyo, Japan). Phenacetin, used as the internal standard (IS), was supplied by Sigma-Aldrich (St. Louis, MO, USA). The reduced nicotinamide adenine dinucleotide phosphate (NADPH) was obtained from Roche Pharmaceutical Ltd (Basel, Switzerland). Cytochrome b5 microsomes and recombinant human CYP2C9 microsomes were kind gifts from Beijing Hospital (Beijing, China). Formic acid (FA, 98% purity) of LC-MS grade was acquired from J&K scientific Ltd. (Shanghai, China). LC-MS grade acetonitrile, methanol and isopropanol were purchased from Merck (Darmstadt, Germany). Ultra-pure water was freshly purified by a Milli-Q A10 System (Milli-pore, Billerica, MA, USA). All of the other chemicals and solvents used were of analytical grade or the highest commercially available grade.

### Conditions for enzymatic activity analysis

Mammalian cells (*e.g.*, COS-7 cells) possess sufficient endogenous NADPH-CYP oxidoreductase and cytochrome b5 to support CYP activities and are much closer to the native state of the CYP protein. However, it is difficult to obtain enough protein to study CYP catalytic activity *in vitro* with these cells (Dai et al., 2013). To overcome this limitation, sf21 insect microsomes expressing 36 CYP2C9 allelic variants were obtained according to our previously established method. Although insect cell systems are practical for protein production, it should be noted that some allelic variants may exhibit lower metabolic activity in Sf21 microsomes compared to intact mammalian cells, as previously observed when comparing data from Sf21 and COS-7 cell systems (Dai et al., 2013). The incubation mixture included CYP2C9*1 or other CYP2C9 variants, purified cytochrome b5 and potassium phosphate buffer (PH 7.4), as well as 10–2,000 µM agomelatine according to our previously reported method 15. The reaction was proceeded and stopped by refrigerating at −80 °C according to our published paper 17. Then, 20 µL phenacetin (1 µg/µL) as an internal standard, and 0.4 mL acetic ether for the extraction. The incubation mixture was centrifuged at 13,000 rpm for 10 min after vortexing. The liquid supernatant was transferred into a clean tube and dried under a nitrogen stream. The resulting residue was dissolved in 100 µL mobile phase and 2 µL of the mixture was injected into the LC-MS/MS system for analysis.

**Figure 2:**
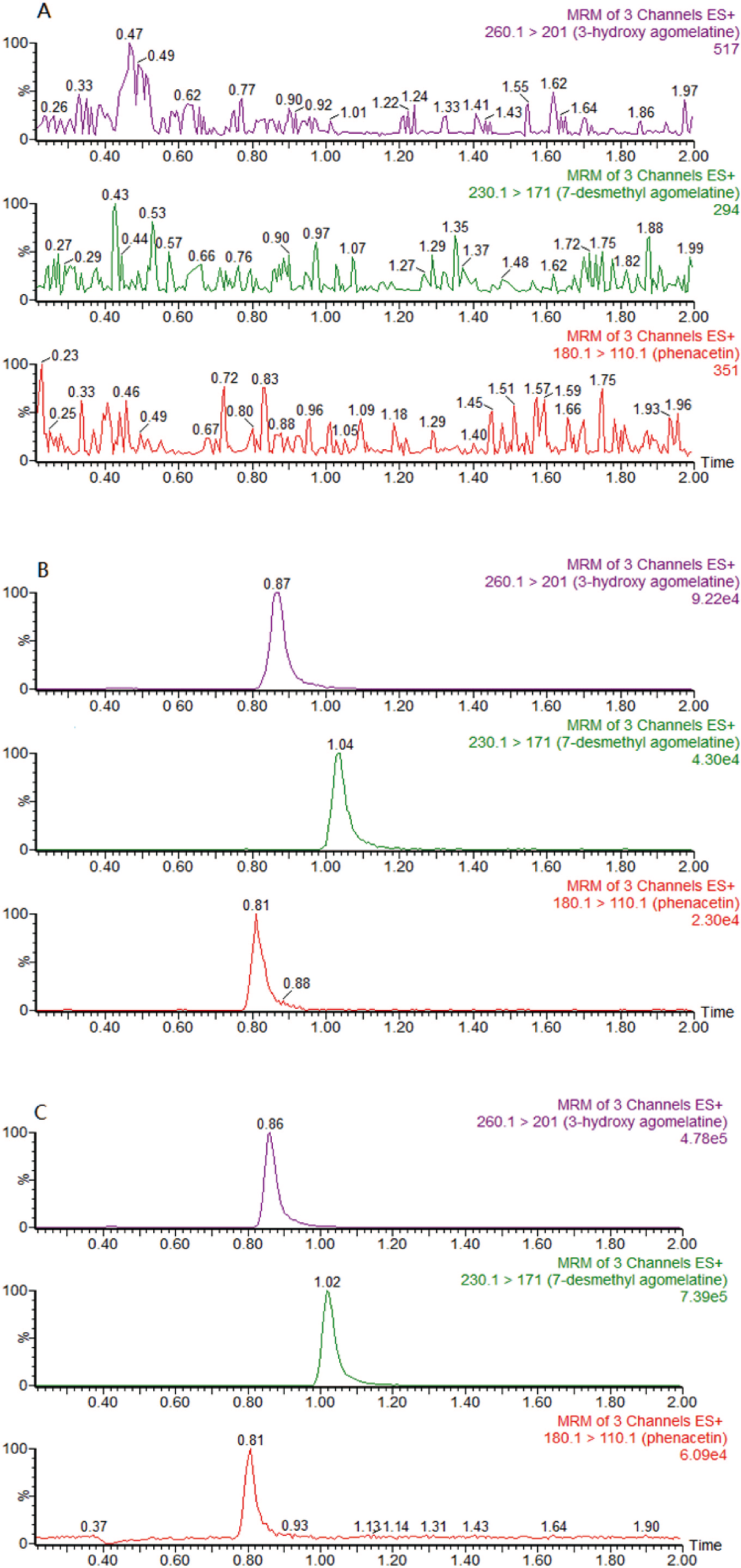
Representative chromatograms of 3-hydroxy agomelatine, 7-desmethyl agomelatine and IS (phenacetin). (A) a blank sample; (B) a blank sample spiked with 3-hydroxy agomelatine, 7-desmethyl agomelatine and IS; (C) an incubation sample.

Quantification determination of the concentrations of agomelatine and its metabolites 3-OH agomelatine and 7-DM agomelatine were performed using UPLC-MS/MS. Chromatographic separations were performed on an ACQUITY UPLC BEH C18 column (2.1 mm × 50 mm, 1.7-µm particle size, Waters Corp.) at 40 °C. The mobile phase consisted of 0.2% formic acid (FA) and methyl alcohol with isocratic elution in the ratio of 45:55, and the elution was performed at a flow rate of 0.3 mL/min. The corresponding parameters of a Waters Xevo TQD triple quadrupole mass spectrometer (Waters Corp., Milford, MA,USA) equipped with an electrospray ionization source (ESI^+^ mode) were showed in Table 1. The retention time of 3-hydroxy agomelatine, 7-desmethyl agomelatine and IS (phenacetin) were 0.87, 1.04 and 0.81 min, respectively Fig. 2.

**Table 1:** The corresponding parameters of UPLC-MS/MS.

Compound	Parent[M+H]^+^ (m/z)	Daughter(m/z)	Cone(V)	Collision(V)
3-OH AGO	260.10	201.00	50	10
7-DM AGO	230.10	171.00	50	15
Phenacetin	180.11	110.12	70	20

### Molecular docking study

Molecular docking analysis was performed to investigate the binding conformations of AGO, 3-OH AGO, and 7-DM AGO within the active sites of human CYP2C9 variants, including CYP2C9*1, *2, *3, *29, and *33. The ligand and protein structures were prepared using standard protocols, and molecular docking was carried out with AutoDock Vina integrated into the PyRx software platform. The resulting docking poses were visualized and analyzed using Discovery Studio 2020 Client to assess key interactions and binding modes.

### Statistical analysis

The enzyme kinetic parameters Michaelis constant (K_m_) and maximum initial velocity (*V*_max_) were estimated by using a software program designed for the non-linear regression analysis of a hyperbolic Michaelis–Menten equation (Prism version 5.0; GraphPad Software, San Diego, CA, USA). The intrinsic clearance (CL_int_) was determined as the ratio of *V*_max_/*K*_m_. Kinetic data for each variant are presented as the mean ± SD of three microsomal preparations derived from separate transfections. One-way analysis of variance (ANOVA) was used for intergroup comparison. Dunnett’s test was used to analyze the differences in catalytic activity between CYP2C9*1 and other CYP2C9 mutants. Statistical analyses were all performed with the Statistical Package for the Social Sciences (version 19.0; SPSS Inc., Chicago, IL, USA), with *p* < 0.05 considered to be statistically significant.

## Results

### Enzymatic studies of agomelatine in CYP2C9 variants

**Figure 3:**
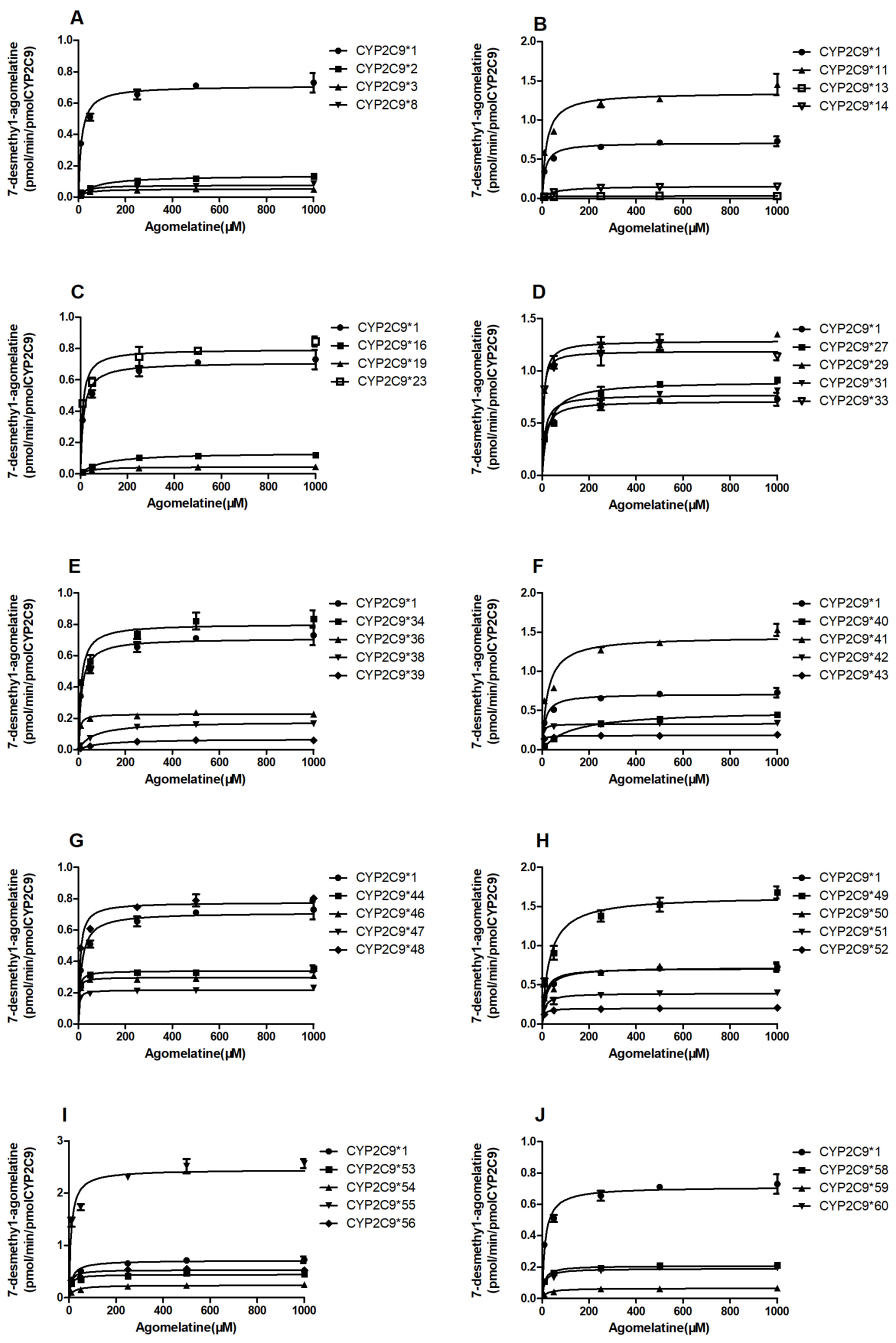
Michaelis–Menten curves of the enzymatic activity of the recombinant wild-type CYP2C9 protein and 36 variants toward AGO 7-demethylation (each point represents the mean ± SD of three separate incubations).

**Figure 4:**
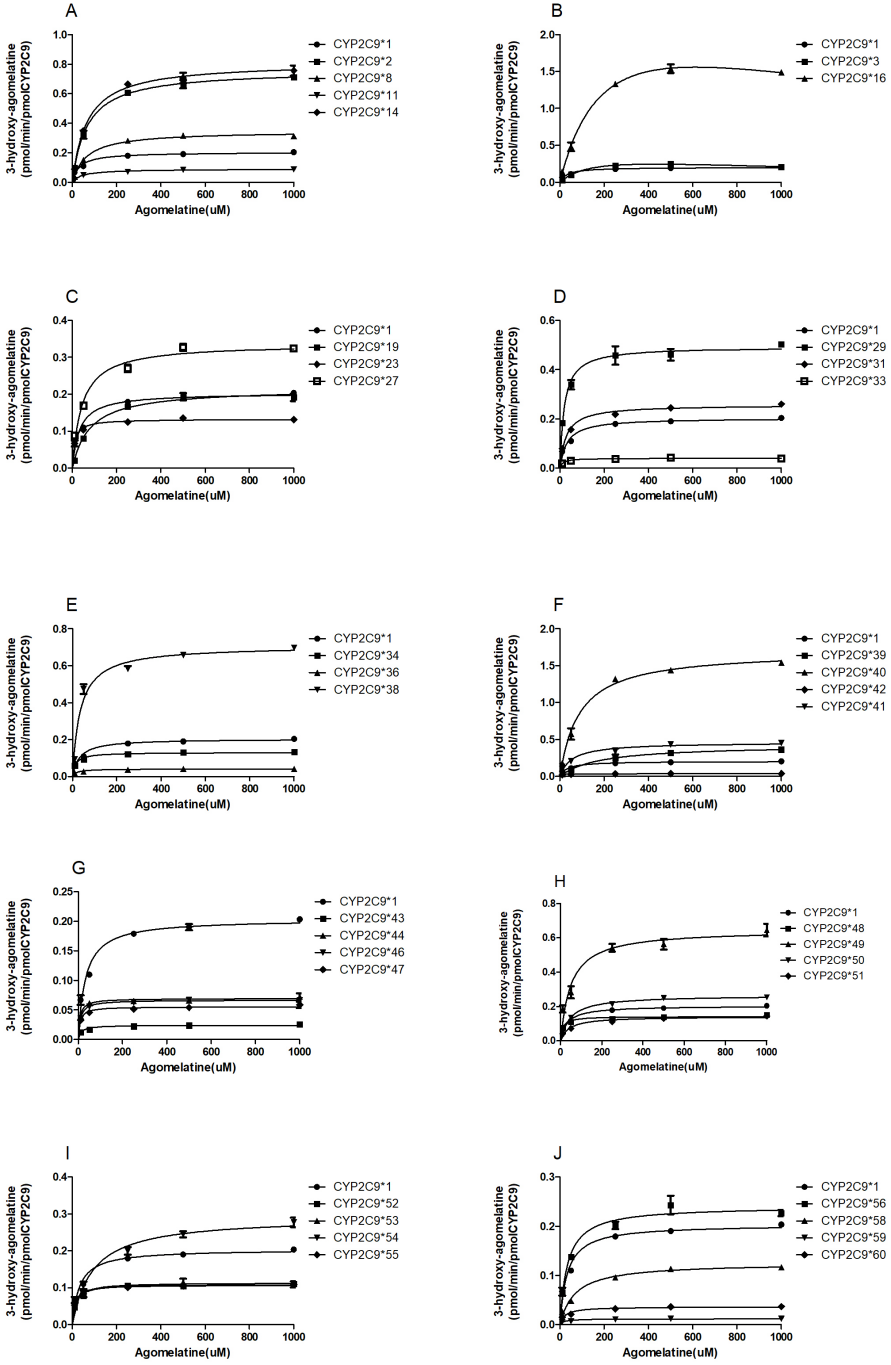
Michaelis–Menten curves of the enzymatic activity of the recombinant wild-type CYP2C9 protein and 36 variants toward AGO 3-hydroxylation (each point represents the mean ± SD of three separate incubations).

In this study, the catalytic activities of wild-type CYP2C9*1 and 36 allelic variants were assessed by using agomelatine as the probe substrate. The intrinsic clearance (*V*_max_/*K*_m_) was used as an evaluation criterion to assess the enzymatic activity of each CYP2C9 allelic variant in agomelatine metabolism *in vitro*. Michaelis–Menten plots towards agomelatine 7-demethylation and 3-hydroxylation by the tested 37 CYP2C9 allelic variants are shown in Figs. 3 and 4, respectively. The corresponding kinetic parameters and relative clearance value compared with wild type (expressed as % relative clearance) are summarized in Tables 2 and 3.

**Table 2:** Enzymatic kinetic parameters of 3-OH AGO generation by 37 different CYP2C9 variants *in vitro* (*n* = 3).

Variants	CYP2C9V_max_ (pmol/min/pmol CYP2C9)	K_m_ (μM)	CL_int_ (μL/min/pmol P450)	Relative clearance (% of wild type)
CYP2C9*1	0.20 ± 0.00	31.96 ± 2.29	0.0064 ± 0.0004	100.00%
CYP2C9*2	0.76 ± 0.03^*^	67.87 ± 12.73^*^	0.0114 ± 0.0018^*^	178.77%
CYP2C9*3	0.44 ± 0.06^*^	165.90 ± 41.86^*^	0.0027 ± 0.0003^*^	42.37%
CYP2C9*8	0.35 ± 0.01^*^	64.69 ± 4.46^*^	0.0054 ± 0.0002^*^	84.16%
CYP2C9*11	0.09 ± 0.00^*^	45.80 ± 10.94	0.0020 ± 0.0006^*^	31.74%
CYP2C9*13	0.07 ± 0.00^*^	85.41 ± 11.93^*^	0.0008 ± 0.0001^*^	12.26%
CYP2C9*14	0.81 ± 0.03^*^	66.17 ± 1.49^*^	0.0123 ± 0.0002^*^	193.19%
CYP2C9*16	2.67 ± 0.05^*^	219.03 ± 21.57^*^	0.0123 ± 0.0010^*^	192.41%
CYP2C9*19	0.22 ± 0.01	80.22 ± 7.79^*^	0.0027 ± 0.0001^*^	42.49%
CYP2C9*23	0.13 ± 0.00^*^	10.78 ± 0.14^*^	0.0123 ± 0.0001^*^	193.64%
CYP2C9*27	0.34 ± 0.00^*^	43.33 ± 1.15^*^	0.0078 ± 0.0001^*^	122.36%
CYP2C9*29	0.49 ± 0.00^*^	19.71 ± 2.63^*^	0.0253 ± 0.0033^*^	399.15%
CYP2C9*31	0.26 ± 0.00^*^	28.52 ± 0.32	0.0090 ± 0.0000^*^	141.48%
CYP2C9*33	0.04 ± 0.00^*^	12.45 ± 0.69^*^	0.0032 ± 0.0002^*^	50.98%
CYP2C9*34	0.13 ± 0.01^*^	14.33 ± 0.94^*^	0.0091 ± 0.0007^*^	143.13%
CYP2C9*36	0.04 ± 0.00^*^	17.40 ± 4.59^*^	0.0025 ± 0.0006^*^	37.11%
CYP2C9*38	0.71 ± 0.02^*^	35.47 ± 4.74	0.0202 ± 0.0024^*^	316.10%
CYP2C9*39	0.43 ± 0.03^*^	179.57 ± 36.41^*^	0.0024 ± 0.0003^*^	38.04%
CYP2C9*40	1.70 ± 0.03^*^	88.04 ± 8.48^*^	0.0194 ± 0.0015^*^	304.40%
CYP2C9*41	0.46 ± 0.01^*^	55.15 ± 9.11^*^	0.0085 ± 0.0015	134.42%
CYP2C9*42	0.04 ± 0.00^*^	6.28 ± 1.10^*^	0.0058 ± 0.0010	89.71%
CYP2C9*43	0.02 ± 0.00^*^	12.71 ± 0.97^*^	0.0019 ± 0.0001^*^	29.68%
CYP2C9*44	0.07 ± 0.00^*^	7.33 ± 0.68^*^	0.0095 ± 0.0007^*^	149.17%
CYP2C9*46	0.07 ± 0.00^*^	9.23 ± 1.36^*^	0.0073 ± 0.0010	113.88%
CYP2C9*47	0.06 ± 0.00^*^	7.72 ± 2.32^*^	0.0075 ± 0.0020	115.40%
CYP2C9*48	0.14 ± 0.01^*^	10.24 ± 2.80^*^	0.0145 ± 0.0037^*^	222.84%
CYP2C9*49	0.65 ± 0.02^*^	50.28 ± 7.36^*^	0.0130 ± 0.0015^*^	204.93%
CYP2C9*50	0.26 ± 0.00^*^	46.63 ± 4.97^*^	0.0057 ± 0.0005	89.44%
CYP2C9*51	0.14 ± 0.00^*^	40.62 ± 4.35^*^	0.0035 ± 0.0004^*^	55.33%
CYP2C9*52	0.11 ± 0.00^*^	14.38 ± 2.96^*^	0.0078 ± 0.0017	121.13%
CYP2C9*53	0.11 ± 0.01^*^	16.63 ± 2.44^*^	0.0069 ± 0.0006	107.74%
CYP2C9*54	0.29 ± 0.01^*^	92.55 ± 18.11^*^	0.0032 ± 0.0005^*^	50.09%
CYP2C9*55	0.11 ± 0.00^*^	10.95 ± 0.69^*^	0.0098 ± 0.0007^*^	154.25%
CYP2C9*56	0.24 ± 0.01^*^	33.35 ± 1.72	0.0072 ± 0.0003^*^	113.65%
CYP2C9*58	0.12 ± 0.00^*^	67.89 ± 4.85^*^	0.0018 ± 0.0001^*^	28.99%
CYP2C9*59	0.01 ± 0.00^*^	20.54 ± 13.05	0.0007 ± 0.0004^*^	10.31%
CYP2C9*60	0.04 ± 0.00^*^	22.84 ± 1.90^*^	0.0016 ± 0.0001^*^	25.66%

**Notes.**

Data are presented as the mean ± standard deviation (SD).

* Represent *P* <0.05 *vs.* wild-type.

**Table 3:** Enzymatic kinetic parameters of 7-DM AGO generation by 37 different CYP2C9 genotypes *in vitro* (*n* = 3).

Variants	V_max_ (pmol/min/pmol CYP2C9)	K_m_ (μM)	CL_int_ (μL/min/pmol P450)	Relative clearance (% of wild type)
CYP2C9*1	0.71 ± 0.03	13.40 ± 2.23	0.0540 ± 0.0078^*^	100.00%
CYP2C9*2	0.14 ± 0.01^*^	73.91 ± 17.04^*^	0.0019 ± 0.0003^*^	3.54%
CYP2C9*3	0.05 ± 0.00^*^	35.05 ± 11.16	0.0016 ± 0.0004^*^	2.93%
CYP2C9*8	0.09 ± 0.00^*^	80.33 ± 0.51^*^	0.0011 ± 0.0000^*^	2.04%
CYP2C9*11	1.35 ± 0.04^*^	18.92 ± 2.07^*^	0.0719 ± 0.0063^*^	133.40%
CYP2C9*13	0.03 ± 0.00^*^	11.01 ± 1.97	0.0028 ± 0.0005^*^	5.19%
CYP2C9*14	0.16 ± 0.01^*^	50.70 ± 4.31^*^	0.0031 ± 0.0001^*^	5.81%
CYP2C9*16	0.14 ± 0.01^*^	94.54 ± 3.66^*^	0.0014 ± 0.0001^*^	2.66%
CYP2C9*19	0.05 ± 0.00^*^	47.97 ± 13.79^*^	0.0010 ± 0.0003^*^	1.83%
CYP2C9*23	0.80 ± 0.02^*^	9.66 ± 1.00	0.0829 ± 0.0089^*^	153.77%
CYP2C9*27	0.90 ± 0.00^*^	26.71 ± 1.98^*^	0.0338 ± 0.0025^*^	62.84%
CYP2C9*29	1.29 ± 0.03^*^	6.40 ± 0.44^*^	0.2016 ± 0.0096^*^	372.64%
CYP2C9*31	0.77 ± 0.00^*^	11.70 ± 0.56	0.0662 ± 0.0032	123.01%
CYP2C9*33	1.19 ± 0.02^*^	4.78 ± 0.80^*^	0.2536 ± 0.0393^*^	464.14%
CYP2C9*34	0.80 ± 0.03^*^	11.68 ± 2.14	0.0702 ± 0.0116	130.61%
CYP2C9*36	0.23 ± 0.00^*^	5.12 ± 1.90^*^	0.0491 ± 0.0191	85.36%
CYP2C9*38	0.18 ± 0.00^*^	66.69 ± 5.71^*^	0.0027 ± 0.0002^*^	5.00%
CYP2C9*39	0.07 ± 0.00^*^	90.53 ± 4.79^*^	0.0008 ± 0.0000^*^	1.41%
CYP2C9*40	0.50 ± 0.00^*^	127.53 ± 10.58^*^	0.0039 ± 0.0003^*^	7.24%
CYP2C9*41	1.45 ± 0.03^*^	24.65 ± 3.93^*^	0.0594 ± 0.0076	109.98%
CYP2C9*42	0.33 ± 0.01^*^	3.51 ± 0.75^*^	0.0968 ± 0.0208^*^	174.28%
CYP2C9*43	0.18 ± 0.00^*^	3.83 ± 0.44^*^	0.0479 ± 0.0051	88.53%
CYP2C9*44	0.34 ± 0.01^*^	3.73 ± 0.34^*^	0.0914 ± 0.0080^*^	169.18%
CYP2C9*46	0.30 ± 0.01^*^	2.84 ± 0.25^*^	0.1053 ± 0.0068^*^	194.70%
CYP2C9*47	0.22 ± 0.00^*^	3.27 ± 0.42^*^	0.0669 ± 0.0072	122.98%
CYP2C9*48	0.78 ± 0.02^*^	7.18 ± 0.49^*^	0.1084 ± 0.0061^*^	200.88%
CYP2C9*49	1.64 ± 0.01^*^	32.63 ± 6.10^*^	0.0514 ± 0.0099	94.62%
CYP2C9*50	0.72 ± 0.02	18.35 ± 3.29	0.0401 ± 0.0059	74.81%
CYP2C9*51	0.39 ± 0.01^*^	11.98 ± 3.32	0.0342 ± 0.0078^*^	63.82%
CYP2C9*52	0.20 ± 0.00^*^	6.78 ± 1.79^*^	0.0312 ± 0.0089^*^	56.35%
CYP2C9*53	0.44 ± 0.02^*^	7.69 ± 1.05^*^	0.0583 ± 0.0060	107.50%
CYP2C9*54	0.24 ± 0.01^*^	20.01 ± 2.73^*^	0.0121 ± 0.0015^*^	22.47%
CYP2C9*55	2.45 ± 0.08^*^	9.28 ± 1.98	0.2705 ± 0.0460^*^	493.19%
CYP2C9*56	0.53 ± 0.01^*^	7.43 ± 1.22^*^	0.0726 ± 0.0095	134.25%
CYP2C9*58	0.21 ± 0.01^*^	10.27 ± 0.64	0.0203 ± 0.0012^*^	37.52%
CYP2C9*59	0.06 ± 0.00^*^	21.52 ± 1.60^*^	0.0030 ± 0.0003^*^	5.59%
CYP2C9*60	0.19 ± 0.01^*^	11.26 ± 1.40	0.0170 ± 0.0018^*^	31.50%

**Notes.**

Data are presented as the mean ± standard deviation (SD).

* Represent *P* <0.05 *vs.* wild-type.

Graphical abstract (For Table of Contents Only).

Nearly all CYP2C9 variants exhibited changed *K*_m_ or *V*_max_ values compared to that of the wild-type enzyme. Consequently, the *V*_max_/*K*_m_ values in most of the tested CYP2C9 isoforms in the present study for both agomelatine 7-demethylation and 3-hydroxylation were significantly altered.

### Agomelatine 3-hydroxylation

Our data revealed that 16 out of the 36 variants exhibited significantly larger *V*_max_ values compared with CYP2C9*1 (*P* < 0.05) in agomelatine 3-hydroxylation, 19 variants showed significantly lower values (*P* < 0.05), while CYP2C9*19 have no significant difference to CYP2C9*1 (Table 2). Meanwhile, 16 of the 36 variants exhibited significantly larger *K*_m_ values compared to CYP2C9*1 (*P* < 0.05). The remaining 15 variants exhibited lower *K*_m_ values compared with CYP2C9*1, while (CYP2C9*11, CYP2C9*31, CYP2C9*38, CYP2C9*56 and CYP2C9*59) exhibited similar *K*_m_ values with CYP2C9*1. Thus, most of the 36 variants exhibited significantly different *V*_max_/*K*_m_ value compared with CYP2C9*1 (*P* < 0.05), except for CYP2C9*41, CYP2C9*42, CYP2C9*46, CYP2C9*47, CYP2C9*50, CYP2C9*52 and CYP2C9*53. Among which, there are 14 variants (CYP2C9*3, CYP2C9*8, CYP2C9*11, CYP2C9*13, CYP2C9*19, CYP2C9*33, CYP2C9*36, CYP2C9*39, CYP2C9*43, CYP2C9*51, CYP2C9*54, CYP2C9*58, CYP2C9*59 and CYP2C9*60) were reduced to 10.31–84.16% compared with the wild type, and the other 14 variants (CYP2C9*2, CYP2C9*14, CYP2C9*16, CYP2C9*23, CYP2C9*27, CYP2C9*29, CYP2C9*31, CYP2C9*34, CYP2C9*38, CYP2C9*40, CYP2C9*44, CYP2C9*e48, CYP2C9*49, CYP2C9*55 and CYP2C9*56) were increased to 113.65%–399.15%.

### Agomelatine 7-demethylation

As shown in Table 3, except for 2C9*50, the V_max_ values of 35 variants basically changed in agomelatine **7-**demethylation compared to that of wild type. Among which, V_max_ values decreased in 24 variant types (CYP2C9*2, CYP2C9*3, CYP2C9*8, CYP2C9*13, CYP2C9*14, CYP2C9*16, CYP2C9*19, CYP2C9*36, CYP2C9*38, CYP2C9*39, CYP2C9*40, CYP2C9*42, CYP2C9*43, CYP2C9*44, CYP2C9*46, CYP2C9*47, CYP2C9*51, CYP2C9*52, CYP2C9*53, CYP2C9*54, CYP2C9*56, CYP2C9*58, CYP2C9*59 and CYP2C9*60) and increased in 11 variant types (CYP2C9*11, CYP2C9*23, CYP2C9*27, CYP2C9*29, CYP2C9*31, CYP2C9*33, CYP2C9*34, CYP2C9*41, CYP2C9*48, CYP2C9*49, and CYP2C9*55). As for the *K*_m_ value, 14 variants (CYP2C9*2, CYP2C9*8, CYP2C9*11, CYP2C9*14, CYP2C9*16, CYP2C9*19, CYP2C9*27, CYP2C9*38, CYP2C9*39, CYP2C9*40, CYP2C9*41, CYP2C9*49, CYP2C9*54 and CYP2C9*59) had increased K_m_ values and 12 variants (CYP2C9*29, CYP2C9*33, CYP2C9*36, CYP2C9*42, CYP2C9*43, CYP2C9*44, CYP2C9*46, CYP2C9*47, CYP2C9*48, CYP2C9*52, CYP2C9*53 and CYP2C9*56) had decreased *K*_m_ values, the remaining 10 variants (CYP2C9*3, CYP2C9*13, CYP2C9*23, CYP2C9*31, CYP2C9*34, CYP2C9*50, CYP2C9*51, CYP2C9*55, CYP2C9*58 and CYP2C9*60) are basically equivalent to the wild type. In terms of *V*_max_/*K*_m_ value, 10 variants (CYP2C9*31, CYP2C9*34, CYP2C9*36, CYP2C9*41, CYP2C9*43, CYP2C9*47, CYP2C9*49, CYP2C9*50, CYP2C9*53, and CYP2C9*56) showed no statistical difference compared with the wild type, nine variants (CYP2C9*11, CYP2C9*23, CYP2C9*29, CYP2C9*33, CYP2C9*55, CYP2C9*48, CYP2C9*e46, CYP2C9*42 and CYP2C9*44) were increased to 133.40%–493.19%. Six variants (CYP2C9*27, CYP2C9*51, CYP2C9*52, CYP2C9*54, CYP2C9*58 and CYP2C9*60) were reduced to 22.47–63.82%, the other 11 genotypes (CYP2C9*2, CYP2C9*3, CYP2C9*8, CYP2C9*13, CYP2C9*14, CYP2C9*16, CYP2C9*19, CYP2C9*38, CYP2C9*39, CYP2C9*40, CYP2C9*59) even down to 1.41%−7.24%.

### Molecular docking study

**Figure 5:**
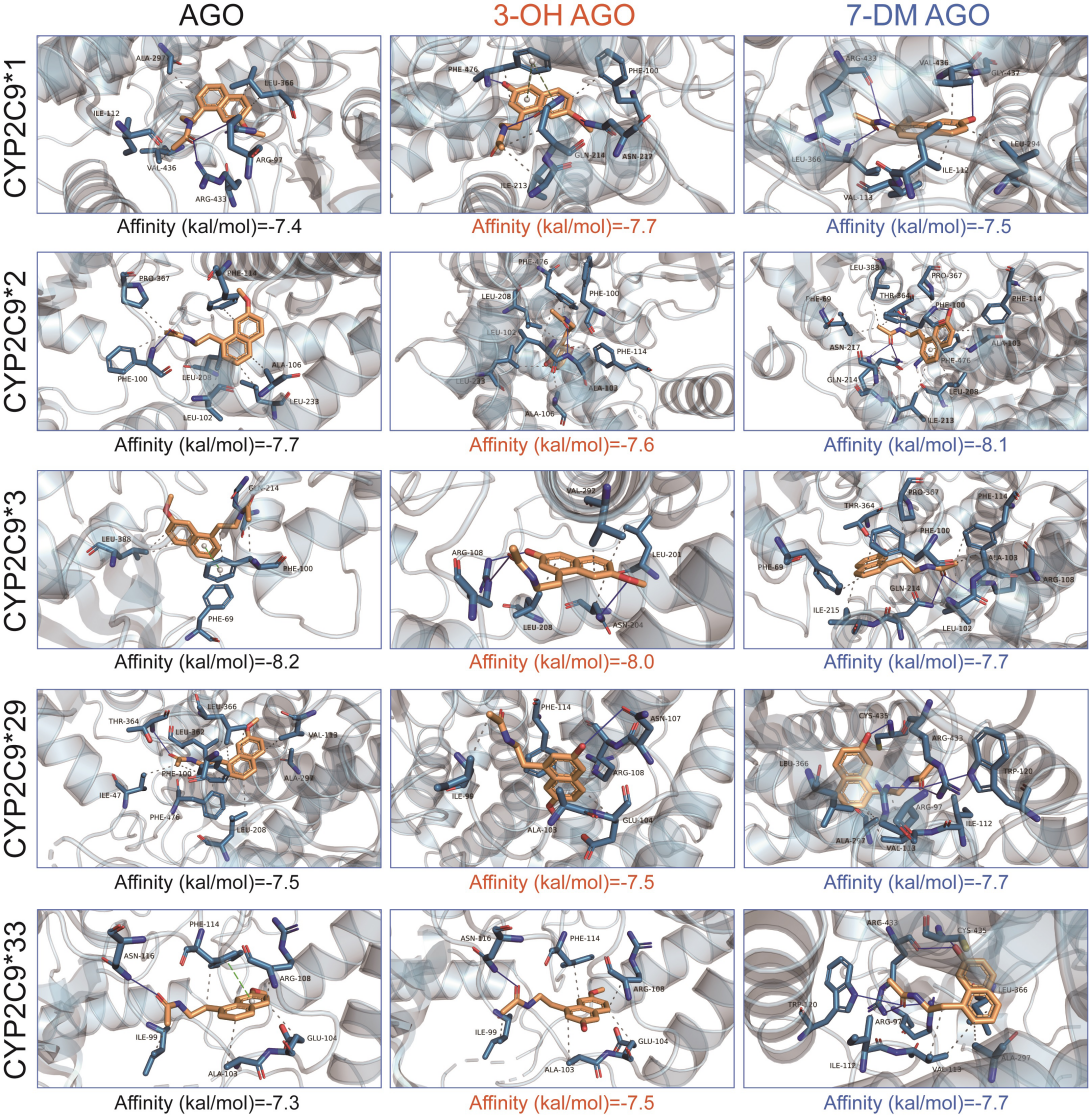
Free binding energies (kcal/mol) and molecular docking analysis of AGO, 3-OH AGO or 7-OH AGO to the active cavities of human CYP2C9*1, CYP2C9*2, CYP2C9*3, CYP2C9*29 and CYP2C9*33, respectively.

The predicted binding conformations and binding energy values of AGO, 3-OH AGO or 7-DM AGO within the active cavities of human CYP2C9*1, CYP2C9*2, CYP2C9*3, CYP2C9*29 and CYP2C9*33, respectively, are presented in Fig. 5.

## Discussion

It is well known that the changes in drug metabolism activity caused by *CYP2C9* gene polymorphism are closely related to adverse drug reactions, as observed with drugs such as phenytoin (Chen et al., 2016), tolbutamide (Hu et al., 2015), and warfarin (Wang et al., 2023). Patients harboring variants with lower catalytic activity than wild-type are at high risk of adverse drug reactions due to elevated drug exposure.

Conversely, individuals with higher metabolic activity may experience subtherapeutic drug concentrations, potentially leading to treatment failure. In this study, agomelatine was incubated individually with recombinant enzyme microsomes expressing 37 *CYP2C9* allelic variants *in vitro*. The enzyme kinetic parameters were studied for wild type and variant types, such as *V*_max_, *K*_m_, and intrinsic clearance (*V*_max_/*K*_m_). The effect of *CYP2C9* gene polymorphism on the metabolism of agomelatine *in vitro* was studied by comparing these kinetic parameters to those of wild type.

**Figure 6:**
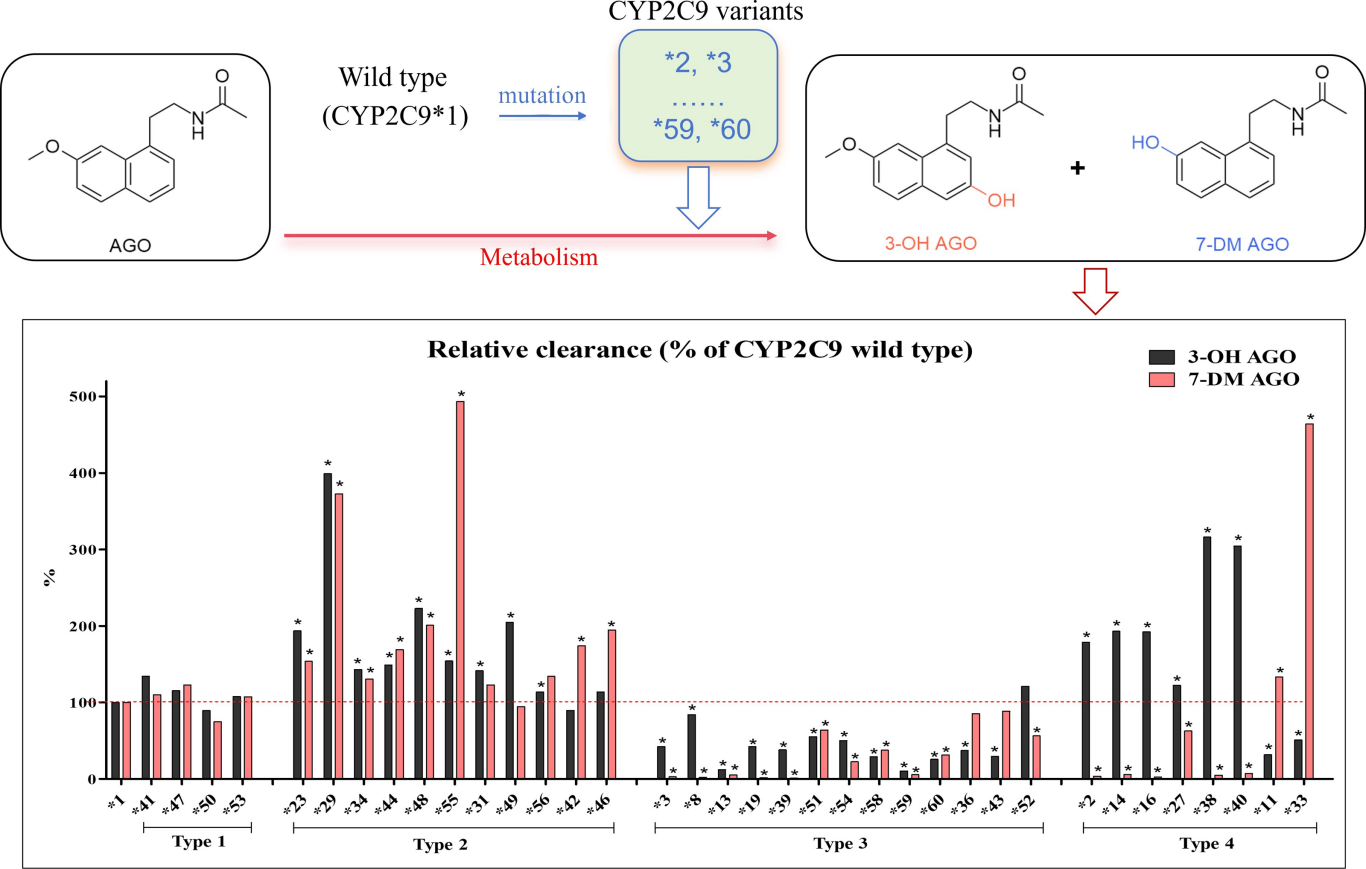
Relative clearance (% of wild type) of generation of 3-OH AGO and 7-DM AGO by 37 different CYP2C9 genotypes *in vitro*.* represents *p* < 0.05*vs.* wild-type.

With respect to the 7-demethylation and 3-hydroxylation of agomelatine *in vitro*, as shown in Fig. 6, the intrinsic clearance values were consistent in most of the identified variants. However, notable discrepancies in clearance were observed for certain variants between the two metabolic pathways. Thus, the 36 variant alleles can be classified into four functional categories. The first type phenomenon (type 1) involved four variants—CYP2C9*41, *47, *50 and *53—that did not exhibit significantly difference in intrinsic clearance compared to the CYP2C9*1, indicating normal metabolic activity. Secondly, 11 variants as category 2—CYP2C9*23, CYP2C9*29, CYP2C9*31, CYP2C9*34, CYP2C9*42, CYP2C9*44, CYP2C9*46, CYP2C9*48, CYP2C9*49, CYP2C9*55 and CYP2C9*56—which exhibit significantly higher intrinsic clearance than the wild type, indicating increased metabolic activity. However, the substrate specificity varies among these variants: CYP2C9*31, CYP2C9*49 and CYP2C9*56 selectively promoted the formation of 3-OH-AGO, while CYP2C9*42 and CYP2C9*46 only enhance the formation of 7-DM-AGO. Considering the possible reduced systemic exposure to agomelatine, a sufficient attention should be paid to carriers of these variant variants in category 2. On the contrary, these variants in third group (type 3) with a decreased relative clearance values (CYP2C9*3, CYP2C9*8, CYP2C9*13, CYP2C9*36, CYP2C9*39, CYP2C9*43, CYP2C9*51, CYP2C9*52, CYP2C9*54, CYP2C9*58, CYP2C9*59 and CYP2C9*60) is metabolically weaker than the wild type, although CYP2C9*36, CYP2C9*43 and CYP2C9*52 only inhibited the production of one of the metabolites significantly, but which might cause accumulated concentrations of agomelatine *in vivo*. Given the hepatotoxicity associated with agomelatine overdose, close attention should be paid to these variants in category 3. The final phenomenon (category 4) is more noteworthy, these variants (CYP2C9*2, *11, *14, *16, *27, *33, *38 and *40) exhibit divergent metabolic activity, wherein the formation of one metabolite is reduced while the other is enhanced. Specifically, they differentially modulate agomelatine metabolism by suppressing one metabolic pathway (either 7-demethylation or 3-hydroxylation) while simultaneously promoting the alternative pathway.

In addition to the wild type allele, CYP2C9*2 and CYP2C9*3 are the most common and well-characterized variant alleles in Caucasian populations. However, CYP2C9*3 is rarely found in Black and Asian populations (Lee, Goldstein & Pieper, 2002; Xie et al., 2002). In Chinese Han population, the *CYP2C9* 3 allele is about 3.3%, with a heterozygous CYP2C9*1/*3 mutation frequency of about 7.6%, and almost no homozygous (CYP2C9*3/*3) mutation exists. CYP2C9*3 has an A>C mutation at position 1,075 in exon 7, which results in the mutation of isoleucine at position 359 of the encoding peptide chain to leucine. Our previous research has demonstrated that CYP2C9*3 is typical defective allele in the Chinese population (Dai et al., 2013; Chen et al., 2016). The same phenomenon occurred in this research, CYP2C9*3 exhibited 42.37% and 2.93% intrinsic clearance of CYP2C9*1 towards agomelatine 3-hydroxylation and 7-demethylation, respectively. Moreover, the K_m_ values of CYP2C9*2 and CYP2C9*3 are significantly larger than those of CYP2C9*1, indicating reduced substrate affinity for agomelatine in the *in vitro* incubation system, which may impair catalytic efficiency and hinder the progression of the enzymatic reaction. However, molecular docking analysis of representative variants in this study (Fig. 5) revealed lower binding energies for agomelatine with CYP2C9*2 (−7.7 kcal/mol) and CYP2C9*3 (−8.2 kcal/mol) compared to CYP2C9*1 (−7.4 kcal/mol). Lower (more negative) binding energy suggests stronger theoretical binding affinity between the enzyme and substrate. This apparent discrepancy between higher Km (lower functional affinity) and more favorable docking energy highlights a mechanistic inconsistency that warrants further investigation.

Our data demonstrate that, in addition to the well-known variants CYP2C9*2 and *3, other allele variants such as *29 and *33 also show extreme differences in metabolic activity toward agomelatine, in the relative rates of 3-hydroxylation *versus* 7-demethylation. For instance, CYP2C9*2 displayed 178.77% of the wild-type intrinsic clearance for 3-hydroxylation, but only 3.54% for 7-demethylation, highlighting a striking divergence in catalytic efficiency between the two pathways. Notably, the molecular docking results for agomelatine and its metabolites with these variants do not fully account for this differential metabolism (Fig. 5). We propose that this phenomenon is best explained by substrate-dependent alterations in multi-catalytic activity within the shared active site of CYP2C9, as previously suggested for other cytochrome P450 enzymes (Tracy et al., 1996). Agomelatine is metabolized at distinct positions by the same CYP2C9 enzyme, enabling both 3-hydroxylation and 7-demethylation. We hypothesize that structural perturbations induced by specific loss-of-function variants disproportionately disrupt the precise active-site architecture required for 3-hydroxylation. These alterations may severely compromise the substrate binding orientation or the proton transfer network essential for the formation of 3-OH-AGO, while having a comparatively lesser impact on the catalytic machinery responsible for 7-demethylation, thereby preserving the formation of 7-DM-AGO. This intra-enzymatic catalytic shift—a differential effect on multiple metabolic pathways within a single variant—has important clinical implications. In individuals carrying such variants, the resulting metabolite profile—characterized by a severe deficit in 3-OH-AGO and relative preservation of 7-DM-AGO—could uniquely influence both therapeutic efficacy and toxicity risk. The therapeutic effects of agomelatine are primarily attributed to its antagonism of 5-HT2C receptors and agonism of melatonin MT1/MT2 receptors. While the precise pharmacological contributions of its metabolites remain incompletely defined, 3-OH-AGO is known to be pharmacologically active, with high affinity for melatonin receptors. A significant reduction in 3-OH-AGO formation due to impaired CYP2C9 activity could therefore diminish the overall melatonergictone in *vivo*. In contrast, 7-DM-AGO is considered largely inactive towards these primary targets (Guardiola-Lemaitre et al., 2014). Consequently, the absence of the contributory effect of 3-OH-AGO might theoretically compromise the full synchronizing effect on circadian rhythms, which is a cornerstone of agomelatine’s mechanism of action. This could manifest clinically as a reduced magnitude of antidepressant response or a delayed onset of action. In such cases, therapeutic efficacy would rely more heavily on the parent drug’s 5-HT2C receptor antagonism, potentially altering the balance of its dual mechanism. Furthermore, agomelatine is associated with a known risk of idiosyncratic hepatotoxicity, the mechanisms of which remain incompletely understood but are suspected to involve the formation of reactive, potentially hepatotoxic metabolites during biotransformation (Dolder, Nelson & Snider, 2008). The metabolic pathway leading to 7-DM AGO involves oxidative N-demethylation, a reaction known to generate reactive aldehyde intermediates. Although 7-DM AGO itself is considered pharmacologically inactive and non-toxic, the metabolic process by which it is formed may contribute to hepatic oxidative or metabolic stress. In summary, a marked reduction in the formation of the active metabolite 3-OH AGO may compromise therapeutic efficacy, while a relative shift toward the 7-demethylation pathway—particularly in the context of elevated systemic exposure to the parent drug—could increase the risk of hepatotoxicity. This dual risk profile highlights the potential clinical value of preemptive CYP2C9 genotyping to identify patients at heightened risk, who may benefit from alternative therapies or intensified safety monitoring.

The cDNA of CYP2C9*59 and *60 have mutations of base pair A>T at 1,300 and T>A at 1,400, respectively. Dai et al. (2015) reported that both mutations are associated with reduced enzyme activity. For example, the exogenous expression of CYP2C9*59 in insect cell microsomes revealed that, despite a similar protein expression level as wild-type CYP2C9, the variant exhibited significantly reduced V_max_, and/or increased K_m_ values toward three CYP2C9-specific substrates (tolbutamide, diclofenac and losartan) (Dai et al., 2015), findings consistent with our observations. For example, as shown in Fig. 6, CYP2C9*60 displayed 25.66% and 31.50% of the intrinsic clearance of CYP2C9*1 for agomelatine 3-hydroxylation and 7-demethylation, respectively. Moreover, the variants CYP2C9*8, *13, 9*19, *36, and *43 also exhibited reduced enzymatic activity toward agomelatine compared to CYP2C9*1 *in vitro*, which aligns with previous results (Chen et al., 2016; Allabi, Gala & Horsmans, 2005).

Another interesting observation is that the intrinsic clearance rates of certain CYP2C9 allelic variants toward agomelatine were far different from those reported for other CYP2C9 substrate drugs. As shown in Table 4, when compared to CYP2C9*1, most tested CYP2C9 variants for the hydroxylation of AGO exhibited different enzymatic activities in contrast to our previous *in vitro* metabolic assessments using phenytoin as a probe substrate (*e.g.*, CYP2C9*2, *11, *14, *16, *23, *29, *31, *34, *38, *41, *42, *44, *47, *48, *50, *51, *52, *53, *55, *56) (Chen et al., 2016). Similarly, for demethylation of AGO, with the exception of CYP2C9*2, *3, *8, *13, *14, *16, *19, *27, *38, *39, *40, *51, *52, *54, the remaining variants also showed different metabolic activities toward carvedilol demethylation in the present study (Pan et al., 2016). In addition, in the study of Dai et al. (2014a) CYP2C9*31 and CYP2C9*47 were predicted to be defective variants (Matimba et al., 2009). However, our results showed that CYP2C9*47 had no significantly different metabolic activity toward agomelatine compared with the wild type, while CYP2C9*31 demonstrates higher intrinsic clearance than CYP2C9*1. Conversely, CYP2C9*54 has been classified as a fast metabolizer variant (Dai et al., 2014b), but showed reduced intrinsic clearance for agomelatine compared to the wild type. These lines of evidence indicated that the differences in these experimental results are mainly due to the structural differences of the different substrates.

**Table 4:** Enzymatic activity of the wild-type and the variants towards agomelatine and phenytoin hydroxylation, agomelatine and carvedilol desmethylation.

Allelic protein	V_max_/K_m_ (% of the wild-type)			
	Agomelatine hydroxylation	Phenytoin hydroxylation	Agomelatine desmethylation	Carvedilol desmethylation
CYP2C9*1	100	100	100	100
CYP2C9*2	178.77^*^	94.8	3.54^*^	67.8^*^
CYP2C9*3	42.37^*^	13.5^*^	2.93^*^	19.1^*^
CYP2C9*8	84.16^*^	6.2^*^	2.04^*^	8.4^*^
CYP2C9*11	31.74^*^	80.7	133.40^*^	33.9^*^
CYP2C9*13	12.26^*^	11.6^*^	5.19%^*^	1.2^*^
CYP2C9*14	193.19^*^	13.3^*^	5.81^*^	18.8^*^
CYP2C9*16	192.41^*^	14.0^*^	2.66^*^	8.7^*^
CYP2C9*19	42.49^*^	3.4^*^	1.83^*^	0.9^*^
CYP2C9*23	193.64^*^	54.8	153.77^*^	14.7^*^
CYP2C9*27	122.36^*^	179.0^*^	62.84^*^	36.1^*^
CYP2C9*29	399.15^*^	115.7	372.64^*^	34.5^*^
CYP2C9*31	141.48^*^	51.4^*^	123.01	31.1^*^
CYP2C9*33	50.98^*^	0.4^*^	464.14^*^	0.3^*^
CYP2C9*34	143.13^*^	72.7	130.61%	27.5^*^
CYP2C9*36	37.11^*^	0.1^*^	85.36	56.0^*^
CYP2C9*38	316.10^*^	75.6	5.00^*^	39.1^*^
CYP2C9*39	38.04^*^	1.1^*^	1.41^*^	6.0^*^
CYP2C9*40	304.40^*^	247.2^*^	7.24^*^	11.1^*^
CYP2C9*41	134.42	365.6^*^	109.98	27.4^*^
CYP2C9*42	89.71	0.1^*^	174.28^*^	9.5^*^
CYP2C9*43	29.68^*^	2.7^*^	88.53	0.2^*^
CYP2C9*44	149.17^*^	58.9	169.18^*^	20.1^*^
CYP2C9*46	113.88	56.9	194.70^*^	22.3^*^
CYP2C9*47	115.40	470.3^*^	122.98	47.8^*^
CYP2C9*48	222.84^*^	108.0	200.88^*^	24.9^*^
CYP2C9*49	204.93^*^	249.8^*^	94.62	59.0^*^
CYP2C9*50	89.44	39.8^*^	74.81	11.0^*^
CYP2C9*51	55.33^*^	478.9^*^	63.82^*^	21.7^*^
CYP2C9*52	121.13	0.2^*^	56.35^*^	1.4^*^
CYP2C9*53	107.74	448.0^*^	107.50	17.9^*^
CYP2C9*54	50.09^*^	54.4^*^	22.47^*^	22.5^*^
CYP2C9*55	154.25^*^	41.8^*^	493.19^*^	5.9^*^
CYP2C9*56	113.65^*^	95.9	134.25^*^	23.3^*^

**Notes.**

* Represent *P* < 0.05 *vs.* wild-type.

The CYP450 gene family is polymorphic and can cause variations in enzyme activity that can decrease, disappear, or increase. In this study, an *in vitro* incubation system using recombinant CYP2C9 enzyme microsomes with agomelatine as the substrate was established to study and evaluate the effect of CYP2C9 gene polymorphism on agomelatine metabolism in Chinese Han population. Our findings indicate that mutations in most CYP2C9 genes lead to changes in the rate of drug elimination. Therefore, when applying agomelatine clinically, close attention should be paid to its blood drug concentration. Timely adjustment to dosage and dosing interval should be made to minimize the incidence of adverse drug reactions (ADRs) and reduce the harm to patients.

## Conclusions

In our study, we focused on 37 CYP2C9 alleles found in the Chinese population and performed a functional characterization of these allelic variants on agomelatine metabolism *in vitro* for the first time. Our results indicate that most CYP2C9 allelic variants exhibit significantly altered enzymatic activities toward both agomelatine 3-hydroxylation and 7-demethylation compared to the wild-type CYP2C9*1. Of which, CYP2C9*3, *8, *13, *19, *36, *39, *43, *51, *52, *54, *58, 9*59 and *60 exhibited markedly reduced metabolic activity toward agomelatine in the present study. Individuals carrying these defective alleles may require lower oral doses of agomelatine in clinical practice to minimize the risk of adverse effects and optimize therapeutic outcomes.

##  Supplemental Information

10.7717/peerj.20973/supp-1 Supplemental Information 1 Raw data

10.7717/peerj.20973/supp-2 Supplemental Information 2 Visual summary of the core findings of the study The genetic polymorphism of Cytochrome P450 2C9 exerts a significant regulatory effect on the in vitro metabolism of agomelatine, and distinct metabolic efficiencies of agomelatine are observed among different genotypic variants.

## Additional Information and Declarations

### Data Availability

The following information was supplied regarding data availability: The raw measurements are available in the Supplementary Files.
