# Supplementary figures and images for: Effect of Cytochrome P450 2C9 genetic polymorphism on agomelatine metabolism in vitro
Source: PeerJ. 2026 Mar 19;14:e20973. doi: 10.7717/peerj.20973 (PMC13006006; doi:10.7717/peerj.20973)

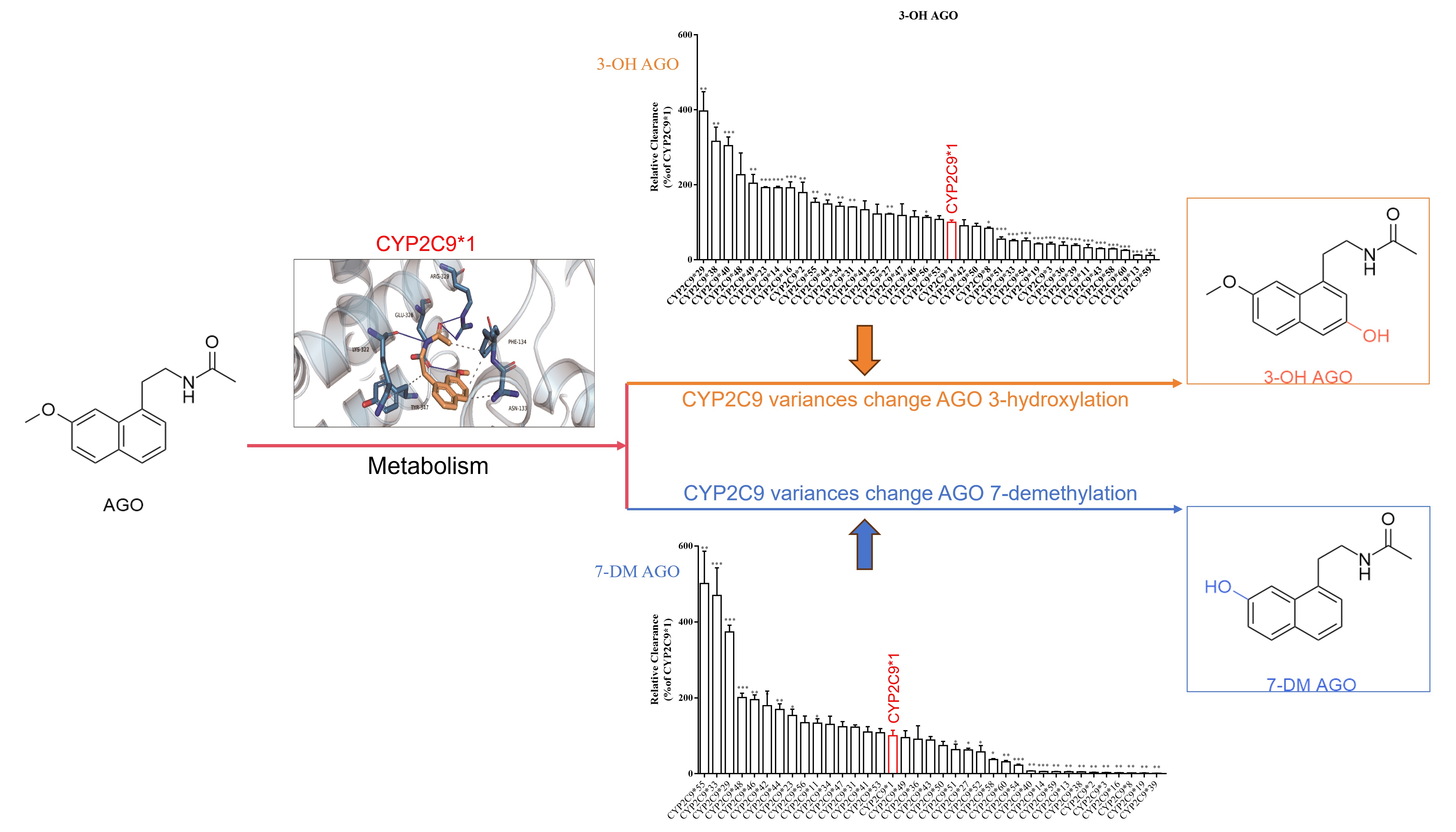

Supplement: Supplemental Information 2 — The genetic polymorphism of Cytochrome P450 2C9 exerts a significant regulatory effect on the in vitro metabolism of agomelatine, and distinct metabolic efficiencies of agomelatine are observed among different genotypic variants. [file peerj-14-20973-s002.jpg]
